# A comprehensive overview of the molecular features and therapeutic targets in *BRAF* ^*V600E*^‐mutant colorectal cancer

**DOI:** 10.1002/ctm2.1764

**Published:** 2024-07-28

**Authors:** Ruiqi Gu, Hongsheng Fang, Renjie Wang, Weixing Dai, Guoxiang Cai

**Affiliations:** ^1^ Department of Colorectal Surgery Fudan University Shanghai Cancer Center Shanghai China; ^2^ Department of Oncology Shanghai Medical College Fudan University Shanghai China

**Keywords:** BRAF, colorectal cancer, molecular features, target therapy

## Abstract

As one of the most prevalent digestive system tumours, colorectal cancer (CRC) poses a significant threat to global human health. With the emergence of immunotherapy and target therapy, the prognosis for the majority of CRC patients has notably improved. However, the subset of patients with *BRAF exon 15 p.V600E* mutation (*BRAF* ^*V600E*^) has not experienced remarkable benefits from these therapeutic advancements. Hence, researchers have undertaken foundational investigations into the molecular pathology of this specific subtype and clinical effectiveness of diverse therapeutic drug combinations. This review comprehensively summarised the distinctive molecular features and recent clinical research advancements in *BRAF*‐mutant CRC. To explore potential therapeutic targets, this article conducted a systematic review of ongoing clinical trials involving patients with *BRAF* ^*V600E*^‐mutant CRC.

## Graphical abstract

- The *BRAF* ^*V600E*^ mutation in colorectal cancer demonstrates distinctive molecular features.
- Colorectal cancer patients with *BRAF* ^*V600E*^ mutation can be classified into two subtypes: BM1 and BM2.
- Various therapeutic combinations, including BRAF, MEK and EGFR inhibitors, improved *BRAF* ^*V600E*^‐mutant colorectal cancer patient outcomes.
- Potential therapeutic targets for the *BRAF* ^*V600E*^ mutation primarily involve the MAPK and PI3K pathways.

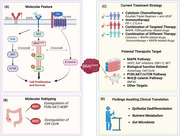

## 1 INTRODUCTION

Colorectal cancer (CRC) is prevalent worldwide, with third highest incidence and fourth highest mortality rates among all tumours, both indicating a rising pattern.^1^ Additionally, a subset of cancers of unknown primary (CUP) exhibits similar molecular and histological features to CRC, known as CUP with a colon‐cancer profile (CUP‐CCP). About 8% of all CUP cases are categorised as CUP‐CCP.^2^, ^3^, ^4^ Despite recent advances in the comprehensive treatment of CRC, long‐term survival beyond 5 years is attained by fewer than 15% of CRC patients with distant metastases.^2^

**FIGURE 1:**
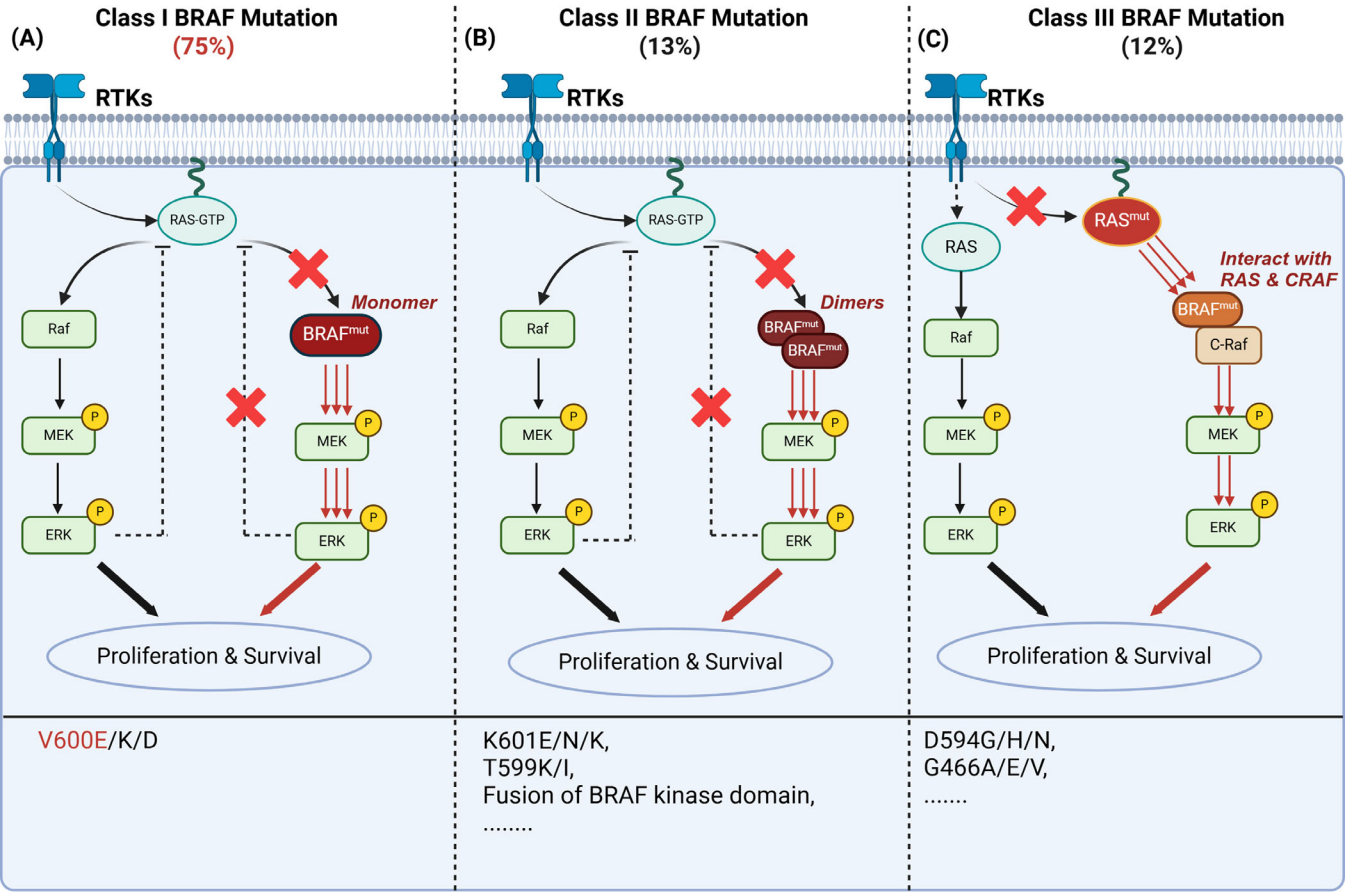
Classification of three types of *BRAF* mutation in colorectal cancer. (A) Class I mutations (V600) is the most common mutation, resulting in the formation of an active monomer with kinase activity. (B) *BRAF* class II mutations forms RAF dimers that are not regulated by RAS. (C) Class III *BRAF* alterations, lead to the formation of heterodimers and need to interact with RAS or CRAF to amplify the effects of upstream signals and carcinogenesis.

In the era of molecular pathology, molecular profiling has become a routine diagnostic approach. This profiling encompasses tests for microsatellite instability (MSI‐H), Human Epidermal Growth Factor Receptor 2(*HER2*) amplification and mutations in Kirsten Rat Sarcoma Viral Oncogene Homolog (*KRAS*) and v‐raf murine sarcoma viral oncogene homolog B (*BRAF*). These analyses play a crucial role in guiding therapeutic decisions and predicting outcomes for individuals affected by CRC. In the case of patients with metastatic colorectal cancer (mCRC), *BRAF* mutations are detected in roughly 5–10%. About 90% of these *BRAF* mutations are localised to codon 600, resulting in the amino acid substitution (*BRAF exon 15 p.V600E*), commonly abbreviated as *BRAF* ^*V600E*^ mutation.^5^ This mutation disrupts the hydrophobic interactions between the P‐loop and the activation segment, consequently promoting the activated conformation of the kinase domain. Such elevation in BRAF kinase activity, spanning from 130 to 700 times that of the wild‐type, leads to prolonged aberrant activation of Mitogen‐activated Protein Kinase (MAPK) pathway and driving the initiation and progression of cancer^6^ (Figure 1).

Pathologically, poor prognostic factors such as mucinous carcinoma, signet ring cell carcinoma, right‐sided colon tumours and sessile serrated adenomas are more commonly observed in patients with *BRAF* ^*V600E*^‐mutations.^7^ *BRAF* mutations are detected in nearly 40% of sporadic MSI‐H CRC, whereas few *BRAF* mutations have been observed in Lynch syndrome patients.^8^ Similarly, in patients with the *BRAF* ^*V600E*^ mutation, approximately 30% of them also exhibit MSI‐H.^9^, ^10^, ^11^ Furthermore, almost 90% of CRC patients with *BRAF* ^*V600E*^ mutation do not harbour Rat Sarcoma Protein (*RAS*) gene mutations, as both mutations activate the same pathway.^12^

*BRAF* ^*V600E*^ mutation serves as an important prognostic factor in late‐stage CRC patients. These individuals exhibit a heightened susceptibility to lymph node and peritoneal metastases, with a lower incidence of pulmonary metastases.^13^, ^14^ Consequently, compared to *BRAF* wild‐type CRC patients, those with *BRAF* ^*V600E*^ mutation experience significantly shorter median overall survival (OS), ranging from 12 to 18 months.^10^, ^15^ With the emergence of tumour immunotherapy and target therapies, the prognosis of mCRC patients has significantly improved. However, the survival benefits for *BRAF* ^*V600E*^‐mutant mCRC patients remain relatively limited.^16^

To gain a comprehensive insight into this distinctive CRC subtype, numerous studies have partially unravelled the molecular alterations and therapeutic targets associated with *BRAF* ^*V600E*^‐mutant CRC. In this review, we systematically examined recent advancements in molecular pathology and treatment approaches for *BRAF* ^*V600E*^‐mutant CRC, including conventional chemotherapy, immunotherapy, and MAPK‐pathway‐related targeted therapy. In our exploration of future treatment strategies, we also conducted a comprehensive review of ongoing clinical trials and preclinical studies focused on the comprehensive treatment of *BRAF* ^*V600E*^‐mutant CRC.

## 2 CLASSIFICATION AND MOLECULAR FEATURE OF *BRAF*‐MUTANT CRC

### 2.1 Classification of *BRAF*‐mutant CRC

As a subtype of the Rat sarcoma viral oncogene homolog (RAF) family, BRAF is a vital kinase and plays a central role in regulating downstream signalling pathways, involving RAS activation and Extracellular Regulated Protein kinases (ERK) phosphorylation^17^ (Figure 1). The mutations of *BRAF* can be classified into three major categories based on mutation sites and biological effects. Class I mutations, primarily involving exon 600, account for nearly 75% of all *BRAF* mutation in CRC.^18^ These mutations result in the formation of an active monomer with kinase activity that is insensitive to upstream signals and downstream negative feedback.^18^, ^19^, ^20^ *BRAF* class II mutations of *BRAF*, which constitute about 13% of *BRAF* mutation in CRC, form RAF dimers that are not regulated by RAS, including *BRAF exon 15 p.K601E/N/T* and fusions involving the *BRAF* kinase domain.^20^, ^21^ Class III alterations, such as *BRAF exon 15 p.D594A/G/H* and *BRAF exon 11 p.G466A/E/V*, also make up approximately 12% of *BRAF* mutation in CRC,^18^, ^20^ leading to the formation of heterodimers. Although the kinase activity of these heterodimers is lower, they can interact with RAS or C‐Raf Proto‐oncogene Serine/threonine‐protein kinase (CRAF) to amplify the effects of upstream signals.^22^ However, class III *BRAF* mutations alone are insufficient to induce carcinogenesis. Thus, additional genetic mutations are required to drive oncogenesis^23^ (Figure 1).

Clinically, CRC patients with *BRAF* ^*V600E*^ and non‐*BRAF* ^*V600E*^ mutations exhibit distinct prognostic features. Although less than 2% of all CRC patients harbour class II and III *BRAF* mutations, individuals within this subgroup exhibit distinctive clinicopathological features, such as left‐sided primary tumours and well‐differentiated histology. Patients with either class II or class III *BRAF* mutations exhibited a more favourable prognosis with an extended median OS of 14.4 months, which is 2 months longer than those with *BRAF* ^*V600E*^mutation.^18^ Although treatment targeting Epidermal Growth Factor Receptor (EGFR) exhibits limited efficacy in treating mCRC harbouring class II *BRAF* mutations, while a majority of CRC patients with class III *BRAF* mutation demonstrate responsiveness.^24^ Notably, CRC patients with non‐*BRAF* ^*V600E*^ mutations derive greater benefit from metastasectomy, achieving a 5‐year OS rate of 89%.^25^, ^26^

### 2.2 Molecular feature and subtyping of *BRAF* ^*V600E*^‐mutant CRC

#### 2.2.1 Molecular feature of *BRAF* ^*V600E*^ in CRC

**FIGURE 2:**
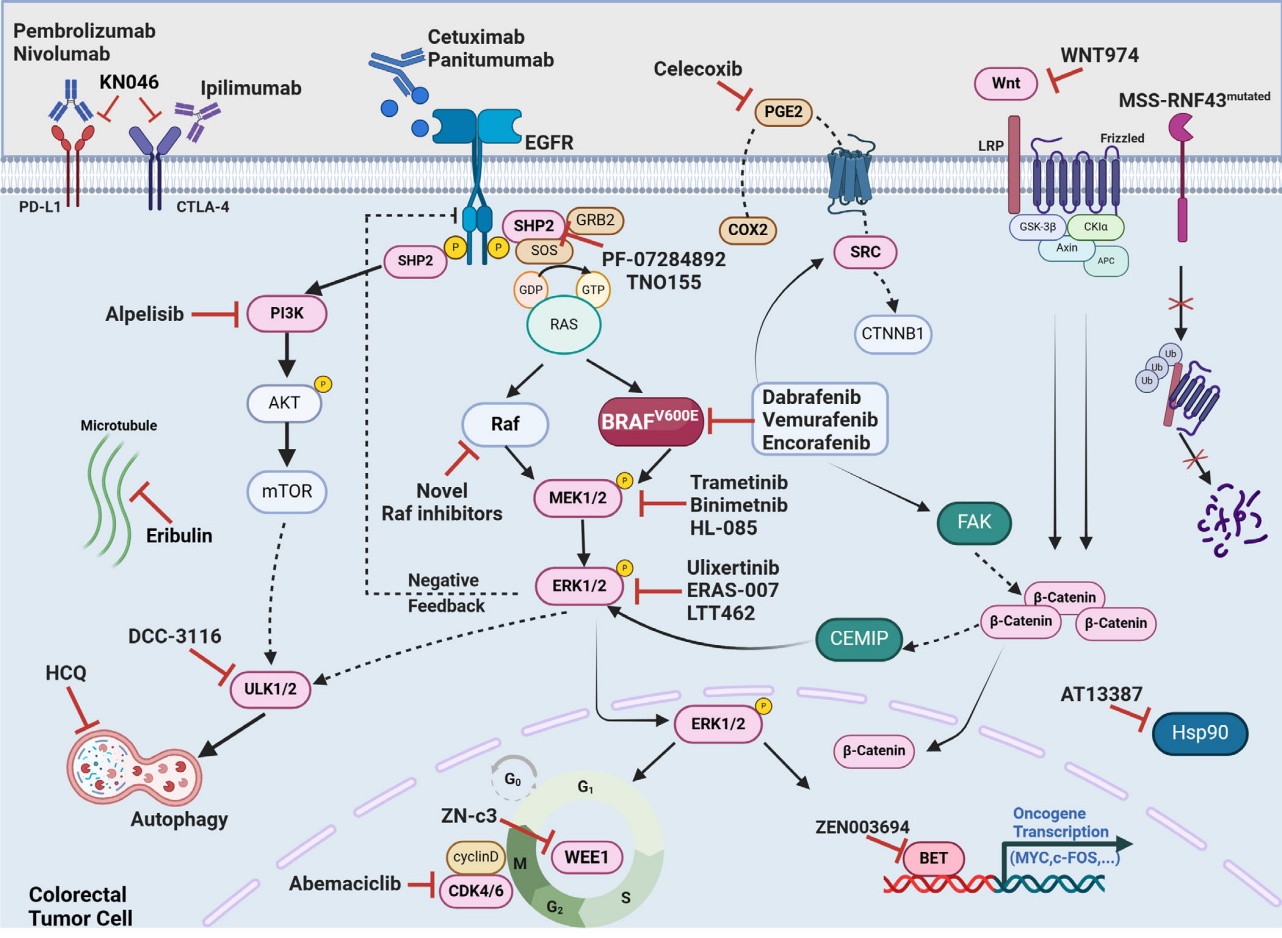
Diagrammatic description of pathways and therapeutic targets in the management of metastatic colorectal cancer (mCRC) featuring the *BRAF* ^*V600E*^ mutation. RAS‐RAF‐ERK pathway, PI3K‐AKT‐mTOR pathway and Wnt/β‐Catenin pathway are the most characteristic molecular pathways in *BRAF* ^*V600E*^ mutation. RAS‐RAF‐ERK pathway, or called MAPK pathway, is directly affected by the overactivated kinase activity of *BRAF* ^*V600E*^, leads to the tumour proliferation. The activation of PI3K‐AKT‐mTOR pathway is also contributed to the acquired resistance of target therapy in *BRAF* ^*V600E*^‐mutant CRC and Wnt/β‐Catenin pathway correlated with both tumourigenesis, metastasis and acquired resistance in *BRAF* ^*V600E*^‐mutant tumour. Those three pathways exhibited extensive crosstalk between each other. Currently, the majority of targeted therapies and clinical trials primarily aims to inhibit three parts: (i) transduction of signalling like SHP2 and Wnt; (ii) specific kinase activities, such as *BRAF* ^*V600E*^, PI3K, MEK and ERK; (iii) downstream biological processes like autophagy, cell cycle regulation and oncogene transcription like WEE1 and BET.

In addition to the MAPK pathway, the *BRAF* ^*V600E*^ mutation induces alterations in various intracellular signalling pathways, including the Phosphoinositide 3‐kinase (PI3K)‐Protein Kinase B (AKT) pathway and the Wingless‐related integration site (Wnt)/β‐catenin pathway. The PI3K‐AKT signalling pathway is also situated downstream of the EGFR^27^, ^28^ (Figure 2). Crosstalk between the PI3K/AKT and MAPK pathways can lead to resistance to BRAF or MAP kinase kinase (MEK) inhibition. The reactivation of EGFR and HER2 may upregulate the PI3K/AKT signalling pathway, resulting in acquired resistance.^29^, ^30^, ^31^ Besides, several studies have confirmed that targeted therapy for *BRAF* ^*V600E*^‐mutant tumours may restore cyclin D1 expression, promoting the cell cycle from the initial growth phase to the synthesis phase, which enables a group of cancer cells to resist cytotoxic effects, thereby generating a resistant tumour cell subpopulation^30^, ^32^ (Figure 2).

In recent years, researchers have also identified a close association between the Wnt/β‐catenin pathway and *BRAF* ^*V600E*^‐mutant CRC. Wnt proteins binding to receptors like Frizzled (Fz) and Lipoprotein receptor‐related protein (LRP) suppresses regulators such as Ring Finger Protein 43 (RNF43), stabilising β‐catenin^33^ (Figure 2). This stabilised β‐catenin can translocate into the nucleus and influence cell death and growth.^34^ In the tumourigenesis of *BRAF* ^*V600E*^‐mutant CRC, the overactivation of Wnt/β‐catenin signalling pathway can impact the differentiation status of intestinal epithelial cells, resulting in aberrant cell proliferation and instability of the intestinal epithelial.^35^

Besides, the Wnt/β‐catenin signalling pathway also contributed to the development of BRAF inhibitor resistance in CRC. Chen and his colleagues discovered that BRAF inhibitors could activate Focal Adhesion Kinase (FAK) in vitro experiments, which can subsequently upregulate the Wnt/β‐catenin signalling pathway.^36^ This pathway also mediates the binding process of Cell Migration‐inducing and Hyaluronan‐binding Protein (CEMIP) with MEK1, enabling the sustained activation of ERK1/2 and MYC (MYC proto‐oncogene)‐driven transcription in MEK inhibitor‐resistant CRC organoids and cell lines (Figure 2 **)**.^37^ Beyond mediating drug resistance, elevated activity of the Wnt/β‐catenin signalling pathway may promote the metastasis of CRC with *BRAF* ^*V600E*^ mutation.^38^

**FIGURE 3:**
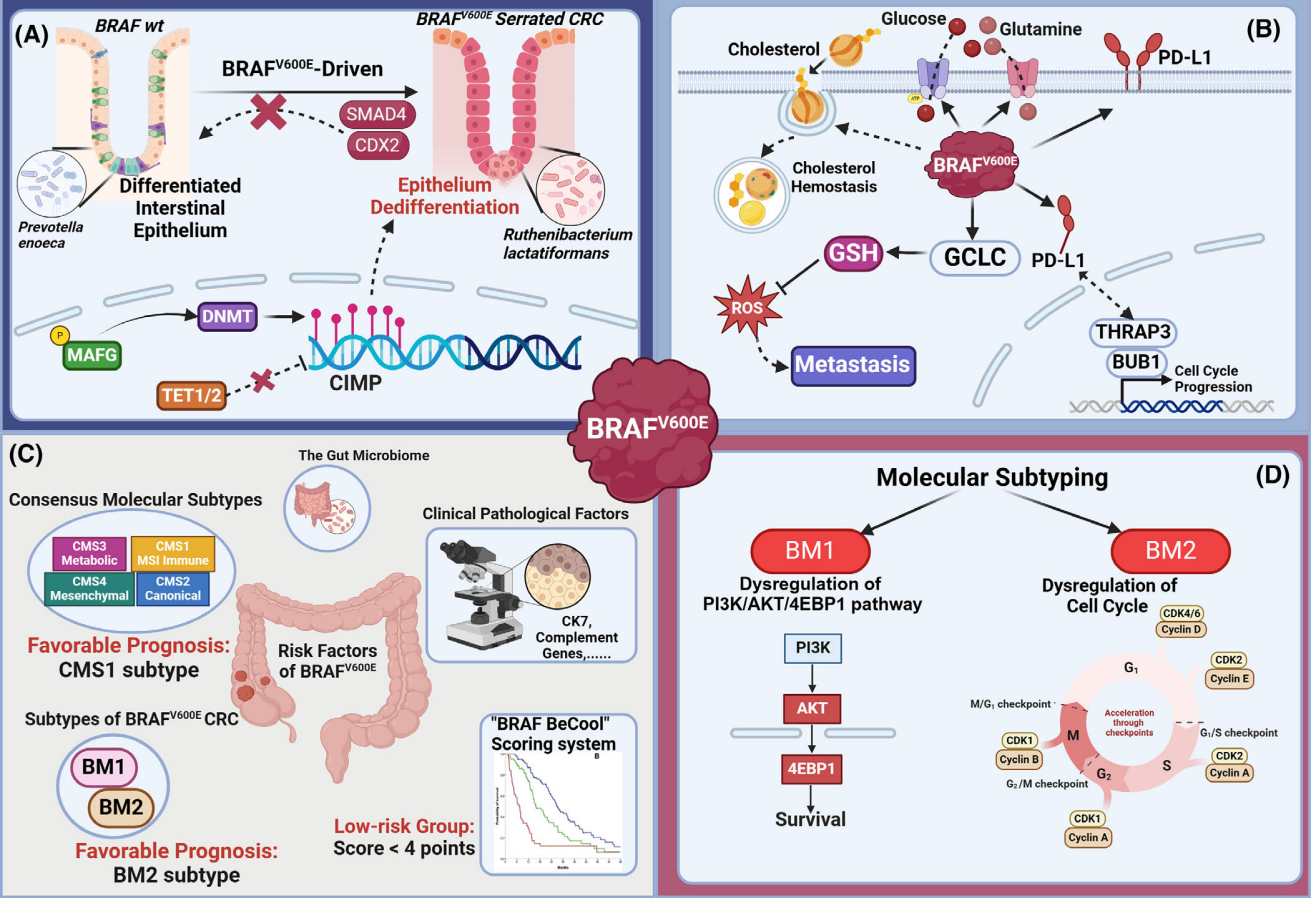
(A) *BRAF* ^*V600E*^‐driven colorectal cancer (CRC) is characterised by dedifferentiation in intestinal epithelial, including the loss function of CDX2, SMAD4 and gut microbiota. *BRAF* ^*V600E*^ also repressed the function of DNA demethylases and caused epigenetic changes in the CpG island. (B) Reprogramming of nutrient metabolism in *BRAF* ^*V600E*^‐mutant CRC. *BRAF* ^*V600E*^ also leads to the elevated expression of PD‐L1 in both the cytoplasm and nucleus of tumour cells. (C) Molecular subtyping and other clinical risk factors of *BRAF* ^*V600E*^‐mutant CRC. (D) Diagrammatic description of the molecular subtyping of *BRAF* ^V600E−^mutant CRC, which is BM1 and BM2. BM1 is characterised by dysregulation of the PI3K/AKT/4EBP pathway, while BM2 manifests dysregulation of the cell cycle, marked by high expression of CDK1 and low levels of cyclin D1.

The presence of *BRAF* ^*V600E*^ leads to elevated Programmed Cell Death Ligand‐1 (PD‐L1) expression on the membrane of CRC cells, which is mainly mediated by MEK and downstream transcription factors like cellular Jun Proto‐oncogene (c‐JUN) and Yes‐associated Protein (YAP).^39^ Furthermore, elevated expression of PD‐L1 was also identified in both the cytoplasmic and nuclear regions of CRC cells and tissues harbouring *BRAF* mutations. Upon interaction with phosphorylated ERK (p‐ERK), PD‐L1 could translocate into the nucleus, leading to the upregulation of the cell‐cycle regulators.^40^ While elevated PD‐L1 levels may promote cell cycle progression and proliferation in *BRAF* ^*V600E*^‐mutant CRC, they also create an opportunity for immunotherapy (Figure 3B).

*BRAF* ^*V600E*^ mutation also mediates a distinctive epigenetic mechanism. *BRAF* can phosphorylate v‐MAF avian musculoaponeurotic fibrosarcoma oncogene homolog G (MAFG), which acts as a transcriptional inhibitor. Phosphorylated MAFG can bind to the promoter region of DNA and recruit corepressive factors such as DNA methyltransferase like DNA (cytosine‐5)‐methyltransferase 3 Beta (DNMT3B). Consequently, this leads to hypermethylation of CpG island methylator phenotype (CIMP)^41^ (Figure 3A). In the mouse model of *BRAF* ^*V600E*^‐driven CRC, a spontaneously acquired aging‐like epigenetic pattern also emerged in the genome.^35^ Meanwhile, the stable expression of *BRAF* ^*V600E*^ in non‐CIMP CRC cell lines can also repress the DNA demethylases of Ten‐eleven Translocation protein family (TET) and initiate hypermethylation of CpG island^42^ (Figure 3A).

#### 2.2.2 Molecular subtyping of *BRAF* ^*V600E*^‐mutant CRC

Based on differential gene expression pattern, *BRAF* ^*V600E*^‐mutant CRC can also be classified into two unique subtypes, designated as BM1 and BM2. BM1 is characterised by activation of the KRAS/AKT pathway and mTOR/4EBP dysregulation, while BM2 manifests dysregulation of the cell cycle, marked by high expression of Cyclin‐dependent kinase 1 (CDK1) and low cyclin D1 expression. Compared to BM2 patients, those with BM1 subtype generally experienced a poorer prognosis.^43^ In consensus molecular subtypes (CMS) of CRC (HR = 0.37, 95% CI 0.19‐0.71, *p* = .003), patients with *BRAF* mutations in CMS1 subtype exhibited a more favourable prognosis (Figure 2D). However, Angerilli's research identified significant heterogeneity within tumour tissues harbouring *BRAF* ^*V600E*^ mutations. Variability in the abundance of infiltrating lymphocytes within the tumour and heterogeneity in CMS classification were observed in different regions of patient's tumour tissue.^44^

In addition to genetic classification, Loupakis' study systematically analysed clinical‐pathological data from 395 patients with the *BRAF* ^*V600E*^ mutation. The study evaluated the impact of various clinical factors on the OS of those patients and established a risk score named ‘BRAF BeCool’. According to the findings of the ‘BRAF BeCool’ analysis, patients classified as low risk (score < 4 points) exhibited an OS of 29.6 months, whereas those high‐risk patients (score > 9 points) demonstrated a significantly reduced OS of 6.6 months (HR for high vs. low risk: 4.72, 95%CI: 2.72−8.20, *p* < .001).^45^ Moreover, elevated expression of Cytokeratin 7 (CK7) and complement genes also served as poor prognostic factors in patients harbouring *BRAF* ^*V600E*^ mutation (Figure 3C).^46^, ^47^

## 3 CURRENT TREATMENT STRATEGY AND ADVANCEMENT IN *BRAF* ^*V600E*^‐MUTANT CRC

### 3.1 First‐line treatment strategy: chemotherapy + anti‐EGFR/VEGF therapy

Similar to the majority of mCRC patients, patients with *BRAF* ^*V600E*^ mutation often rely on chemotherapy as the cornerstone of adjuvant therapy. Despite receiving first‐line treatment with standard two‐drug chemotherapy regimens, including 5‐fluorouracil (5‐FU), leucovorin and oxaliplatin (FOLFOX); 5‐FU, leucovorin and irinotecan (FOLFIRI); and capecitabine and oxaliplatin (CAPEOX), these patients exhibited a median PFS time of merely 6.3 months (*n* = 69 patients; 95% CI: 4.9‐7.7 months).^48^

Diverging from systemic therapy, target therapy can identify and focus on tumour cells through the recognition of tumour‐specific cell membrane receptors or signalling pathway. In recent years, clinicians have explored the incorporation of target therapy into conventional first‐line chemotherapy in treating mCRC patients.

Target therapies in CRC, currently used in clinical practice, include anti‐angiogenic agents and those targeting the EGFR‐mediated MAPK pathway. As mentioned before, *BRAF* ^*V600E*^ mutation activates downstream signalling molecules in the EGFR pathway, thereby promoting tumour proliferation and metastasis. Similarly, clinical trials have also shown that patients with *BRAF* ^*V600E*^ mutation often do not benefit from anti‐EGFR treatments.^49^, ^50^ In a prospective clinical trial, which added anti‐EGFR regimen to FOLFIRI, patients with *BRAF* ^*V600E*^ mutation did not derive extra benefits from anti‐EGFR treatment (HR = 0.69; 95% CI: 0.32−1.49; *p* = .3).^51^

To extend the PFS of *BRAF* ^*V600E*^‐mutant mCRC, researchers have also explored the addition of anti‐angiogenic agents to chemotherapy. In the retrospective subgroup analysis of AGITG MAX clinical trial, researchers found that patients with *BRAF* ^*V600E*^ mutation treated with capecitabine and bevacizumab had a PFS time similar to that of *BRAF* wild‐type patients (HR = 0.80; 95% CI = 0.54 to 1.18), indicating that *BRAF* mutations have little impact on the efficacy of anti‐angiogenic drugs.^52^ Similar results were validated in multiple Phase III clinical trials using bevacizumab.^53^

For mCRC patients with a favourable Eastern Cooperative Oncology Group (ECOG) performance status (score of 0 or 1), an intensified chemotherapy regimen consisting of 5‐FU, leucovorin, oxaliplatin and irinotecan (FOLFOXIRI) in combination with bevacizumab is recommended as the preferred first‐line treatment.^54^ In a Phase III clinical trial (TRIBE) comparing FOLFIRI +bevacizumab versus FOLFOXIRI + bevacizumab as first‐line chemotherapy regimens, 28 patients with *BRAF* mutation were enrolled.^55^ The results revealed that mCRC patients with *BRAF* mutation undergoing triplet chemotherapy combined with bevacizumab achieved a median OS of 19 months, compared to the median OS of only 10.7 months in patients receiving the standard two‐drug chemotherapy (HR = 0.54; 95% CI: 0.24‐1.20)^56^ (Table 1). In the Phase II clinical trial (FIRE‐4.5), researchers enrolled 109 patients with *BRAF* ^*V600E*^ mutation, randomly assigning them to either FOLFOXIRI + bevacizumab or FOLFOXIRI + cetuximab. The results indicated that patients receiving triplet chemotherapy + bevacizumab had an objective response rate (ORR) of 51%, whereas the ORR for those in the triplet chemotherapy + cetuximab group was 41% (odds ratio = 1.87; 80% CI, 1.06 to 3.52; *p* = .92). Regarding median PFS statistics, the bevacizumab group achieved 10.7 months, compared to 6.7 months in the cetuximab group (HR = 1.89; *p* = .006)^57^ (Table 1). In those studies, patients with *BRAF* mutation derived clear survival benefits from the intensified chemotherapy combined with bevacizumab. However, in a subgroup analysis of the subsequent Phase III clinical trial (TRIBE2), patients with *BRAF* mutation did not derive additional survival benefits from the regimen of FOLFOXIRI + bevacizumab, compared to those who received FOLFOX+ bevacizumab (HR for PFS = 1.23, 95% CI 0.72‐2.09; HR for OS = 1.35, 95% CI 0.27‐2.30).^58^

**TABLE 1:** Summary of the significant clinical trials of targeted therapy, immunotherapy and chemotherapy for *BRAF* ^*V600E*^‐mutant metastatic colorectal cancer.

Treatment strategy	Drug schedule	Patients (*n*)	ORR (%)	mPFS (months)	mOS (months)
First‐line chemotherapy + target therapy	‐FOLFOXIRI + bevacizumab^56^	16	56	7.5	19
	‐FOLFOXIRI + bevacizumab^57^	35	51	10.7	17.1
	‐FOLFOXIRI + Cetuximab^57^	72	41	6.7	12.9
	‐FOLFOXIRI + bevacizumab^12^	69	52	6.6	13.4
	‐FOLFOX + bevacizumab^12^	69	31	6.1	12.8
	‐FOLFIRI + bevacizumab^56^	12	42	5.5	10.7
	‐FOLFOX/FOLFIRI + bevacizumab^59^	10	/	9	/
	‐FOLFOXIRI + bevacizumab^59^	12	/	10.6	/
BRAF inhibitor + anti‐EGRF therapy	‐Encorafenib + Cetuximab^60^	220	20	4.2	8.4
	‐Encorafenib + Cetuximab^61^	97	17	4.6	7.2
	‐Vemurafenib + panitumumab^62^	15	13	3.2	7.6
	‐Dabrafenib + panitumumab^63^	20	10	3.5	∖
	‐Vemurafenib + erlotinib^64^	32	16	3.9	6.3
BRAF inhibitor + MEK inhibitor	‐Dabrafenib + trametinib^65^	43	14	3.5	∖
BRAF inhibitor + anti‐EGRF therapy + MEK/PI3K inhibitor	‐Dabrafenib + trametinib + panitumumab^63^	91	21	4.2	9.1
	‐Encorafenib + Cetuximab + binimetinib^60^	224	26	4.3	9
	‐^*^Encorafenib + binimetinib + Cetuximab^66^	95	47.4	5.8	18.3
	‐Encorafenib + Cetuximab + alpelisib^67^	28	18	4.2	∖
	‐Encorafenib + Cetuximab + alpelisib^68^	52	27	5.4	15.2
BRAF inhibitor + anti‐EGRF therapy + cytotoxicity chemotherapy	‐Vemurafenib + Cetuximab + irinotecan^69^	106	16	4.4	∖
	‐Vemurafenib + Cetuximab + FOLFIRI^70^	21	81	9.7	15.4
Immunotherapy	‐^*^Pembrolizumab^71^	153 (23% with *BRAF* ^*V600E*^)	83	16.5	∖
	‐Pembrolizumab^72^	14	42	∖	∖
	‐Ipilimumab + Nivolumab^73^	119 (25% with *BRAF* ^*V600E*^)	65	∖	∖
	‐^*^Ipilimumab + Nivolumab^74^	45 (38% with *BRAF* ^*V600E*^)	69	∖	∖
Immunotherapy + MAPK‐related targeted therapy	‐Sparatlizumab + dabrafenib + trametinib^75^	37	24	4.3	∖
	‐Nivolumab + encorafenib + Cetuximab^76^	23	45	7.3	∖

* Using this regimen as the first‐line treatment.

ORR: objective response rate; mPFS: median progression‐free survival; mOS: median overall survival.

In addition to prospective clinical trials, several retrospective studies have also attempted to explore the pros and cons of triplet versus doublet chemotherapy. The result of a meta‐analysis demonstrated that, in comparison to two‐drug chemotherapy regimens (FOLFOX or FOLFIRI + bevacizumab), FOLFOXIRI + bevacizumab did not significantly improve median OS for patients with *BRAF* mutation (13.6 months vs. 14.5 months, HR = 1.11, 95% CI: 0.75‐1.73).^77^ In a retrospective European study (CAPSTAN CRC), 255 patients with *BRAF* ^*V600E*^ mutation were analysed, with 68.7% of patients receiving first‐line treatment involving chemotherapy combined with targeted therapy. Among them, patients opting for FOLFOX + cetuximab and FOLFOXIRI + bevacizumab accounted for 27.1% each. Regardless of the regimen, the median PFS is approximately 6 months for *BRAF*‐mutated patients (Table 1). Among those receiving triplet chemotherapy plus targeted therapy, the ORR is 52.5% (95% CI 37.0%−68.0%), while for those undergoing doublet chemotherapy plus targeted therapy, it is 31.4% (95% CI 23.0%−39.7%).^12^ However, triplet chemotherapy is also associated with a higher incidence of side effects.

Recently, in a Phase III clinical trial (CAIRO5) targeting unresectable colorectal cancer liver metastasis (CRLM), a total of 29 patients with *BRAF* ^*V600E*^ mutation were enrolled, with 22 having the primary tumour in the right colon and 7 in the left colon. The results indicated that patients with *BRAF*‐mutant CRC originating from the right colon benefited more from intensified chemotherapy plus bevacizumab, with a median PFS of 10.6 months. In contrast, patients receiving the conventional two‐drug regimen plus bevacizumab had a median PFS of 9.0 months (HR = 0.76, 95% CI = 0.60−0.98, *p* = .032). However, the survival benefit was not significant for patients with the primary tumour in the left colon^59^ (Table 1). Notably, the evidence of enhanced survival outcomes for *BRAF*‐mutant CRC patients is still questionable, as patients with *BRAF* ^*V600E*^ mutation constituted only 5% of all participants in this trial. Based on the comprehensive results of the mentioned trials, the ESMO Clinical Practice Guidelines still recommend dual‐agent cytotoxic chemotherapy ± bevacizumab as the first‐line treatment for patients with *BRAF* ^*V600E*^‐mutant CRC. Triple‐agent cytotoxic chemotherapy ± bevacizumab is viewed as an alternative option specifically for those with CRC harbouring *BRAF* ^*V600E*^ mutation in the right colon.^78^ In contrast, the NCCN guidelines commonly recommend a first‐line treatment regimen of triplet chemotherapy combined with bevacizumab for mCRC patients with a relatively good physical condition. However, for the subgroup of patients harbouring the *BRAF* ^*V600E*^ mutation, no specific recommendations are provided.^79^

To further enhance the efficacy of first‐line treatments, various biomarkers have been explored to identify potential beneficiaries. Among these, microRNAs (miRNAs) show considerable promise.^80^ In patients with mCRC, serum miR‐19a can effectively distinguish individuals who are resistant to FOLFOX, and elevated levels of miR‐126 in patients’ plasma are associated with resistance to bevacizumab.^81^, ^82^ Additionally, increased expression of miR‐31, miR‐100 in tumour tissue, as well as downregulation of miR‐7, correlate with resistance to cetuximab.^83^, ^84^ However, the predictive value of miRNAs necessitates validation through large‐scale clinical trials in the future.

### 3.2 Combination of MAPK‐pathway‐related targeted therapy

#### 3.2.1 BRAF inhibitor combined with anti‐EGFR therapy

Although BRAF inhibitors show excellent therapeutic efficacy in *BRAF* ^*V600E*^‐mutant melanoma, CRC patients with *BRAF* ^*V600E*^‐mutation receiving monotherapy with BRAF inhibitors only exhibit an ORR of 5%, with a median PFS of only 2.1 months.^85^

The rationale behind this phenomenon is that BRAF inhibitors transiently suppress p‐ERK, but the reduction in ERK phosphorylation levels leads to the rapid reactivation of EGFR‐mediated RAS and C‐RAF. The reactivation of EGFR significantly attenuates the activity of BRAF inhibitors (Figure 2).^86^ Therefore, researchers attempted a combination therapy using EGFR‐monoclonal antibody and BRAF inhibitors for *BRAF* ^*V600E*^‐mutant patients. In Phase III randomised controlled trials (RCT) and real‐world studies involving *BRAF* ^*V600E*^‐mutant patients who have progressed after first‐line treatment, the ORR for the dual‐targeted therapy (BRAF inhibitors and anti‐EGFR drugs) ranged around 15−20%, and the median PFS was approximately 3–4 months. Thus, this doublet regimen demonstrates a remarkable efficacy compared to the standard regimen of dual‐drug chemotherapy plus cetuximab^60^, ^61^, ^63^, ^87^, ^88^ (Table 1). The combination of encorafenib and cetuximab has been recommended in the second‐line therapy in the latest NCCN guideline.^79^

To identify potential beneficiaries, Ros's research team identified the utility of employing the plasmatic *BRAF* allele fraction (AF) as a predictive marker for the effectiveness of this therapy. Notably, patients exhibiting a high *BRAF* AF demonstrated a significantly inferior PFS compared to their low‐*BRAF* AF counterparts (HR = 2.97, 95% CI 1.55‐5.69; *p* = .001). Intriguingly, among the cohort of high‐*BRAF* AF patients, there was a discernible therapeutic advantage in response to MAPK‐targeted therapy in contrast to their low‐*BRAF* AF counterparts.^89^

Apart from monoclonal antibodies targeting the cell membrane‐bound EGFR, small molecular receptor tyrosine kinase (RTK) inhibitors, including Erlotinib, can also competitively bind to the intracellular catalytic domain of RTK, disrupting intracellular signalling of the EGFR pathway.^90^ In a Phase II clinical trial (EVICT) that enrolled 32 *BRAF* ^*V600E*^‐mutant patients, 16% of patients receiving treatment combining Erlotinib with BRAF inhibitors demonstrated partial response (PR), with a median PFS of 3.9 months (95% CI, 1.8‐5.4)^64^ (Table 1). Compared to patients treated with the regimen of encorafenib + cetuximab, the efficacy of EVICT appears to be generally comparable in terms of median PFS (3.9 months versus 4 months).

Regarding side effects, patients exhibited good tolerability to the dual‐targeted treatment, and skin‐related adverse effects were relatively minor.^62^ The predominant adverse effects include acneiform dermatitis, diarrhoea and nausea. Among the cohort of 216 patients treated with encorafenib + cetuximab, the incidence of adverse effects exceeding grade 3 was observed to be 12.9% (28/216).^91^ According to the findings of the mentioned clinical trial, the dual‐targeted therapy, addressing both EGFR and BRAF, may be suitable for those patients in good health with limited prior treatments.

#### 3.2.2 BRAF inhibitor combined with MEK inhibitor

When blocking the upstream EGFR in the MAPK signalling pathway, the amplification of downstream MEK poses a risk of acquired resistance in the treatment of patients with *BRAF* ^*V600E*^‐mutant mCRC.^92^ Henceforth, researchers have posited a therapeutic strategy involving concurrent inhibition of BRAF and MEK. Trametinib and binimetinib are two commonly used molecular inhibitors that target MEK protein kinase activity.^93^ The concurrent use of encorafenib and binimetinib has been shown to significantly extend PFS and reduce the probability of acquired resistance in patients with advanced melanoma harbouring *BRAF* ^*V600E*^ mutation.^94^ Therefore, researchers have also explored similar approaches in treating *BRAF* ^*V600E*^‐mutant mCRC patients.

The result of an exploratory trial, involving 43 patients with *BRAF* ^*V600E*^‐mutant mCRC, indicated that after receiving dual‐targeted therapy with BRAF and MEK inhibitors, 14% of patients achieved a response (including PR and complete response) and the median PFS was 3.5 months^65^ (Table 1). These results suggest a certain level of activity for this doublet treatment in patients with *BRAF* ^*V600E*^‐mutant mCRC, with efficacy comparable to the combination of EGFR monoclonal antibodies and BRAF inhibitors. However, when compared to the ORR rate exceeding 50% in *BRAF* ^*V600E*^‐mutant melanoma patients, the ORR of this regimen remains suboptimal, falling below 15%.

To delineate the potential beneficiaries of MEK inhibitor, molecular profiling and clinical subgroup analysis from the BEACON trial indicates that patients with tumours exhibiting CMS4 and/or BM1 subtypes are associated with the augmented response rates upon the addition of MEK inhibitors (CMS4: 33.3%, 95% CI: 21.7%−46.7%; BM1: 33.3%, 95% CI: 21.4−47.1%).^95^ Moreover, among patients characterised by elevated baseline C‐reactive protein (CRP) levels (HR: 0.76, 95% CI: 0.54– 1.06), an ECOG performance status of 1 (HR: 0.81, 95% CI: 0.59−1.11), incompletely resected primary tumours (HR: 0.80, 95% CI: 0.56−1.14), and involvement of ≥3 organs (HR: 0.69, 95% CI: 0.49−0.96), additional inhibition of MEK emerges as a more favourable therapeutic choice.^96^

#### 3.2.3 BRAF, MEK, EGFR

Despite the superior efficacy of doublet therapy targeting the MAPK pathway in the treatment of patients with *BRAF* ^*V600E*^ mutation, it still falls short of the efficacy achieved with the combination of BRAF and MEK inhibitors in *BRAF* ^*V600E*^‐mutant metastatic melanoma, which has demonstrated an ORR of 67% and a PFS of 9.3 months.^97^ Therefore, researchers have attempted triplet combinations involving EGFR, BRAF and MEK to enhance the effectiveness of targeted therapy.^98^ In early exploratory trials, patients receiving dabrafenib + panitumumab + MEK inhibition with trametinib demonstrated an ORR of 21%, while the ORR for dual‐targeted therapy with dabrafenib + panitumumab was 10%^63^ (Table 1). To further verify the efficacy of triplet‐targeted therapy, the multicentre Phase III (BEACON CRC) trial enrolled 665 patients with *BRAF* ^*V600E*^‐mutant CRC who had progressed after first‐line chemotherapy. These patients were randomised to the triplet‐therapy group, doublet‐therapy group (encorafenib + cetuximab), and FOLFIRI group for further treatment.^60^ Subsequent efficacy analysis of BEACON trial revealed an ORR of 26.8% (95% CI: 21.1%−33.1%) for the triplet‐therapy group, 19.5% (95% CI 14.5%−25.4%) for the doublet‐therapy group and 1.8% (95% CI: 0.5%−4.6%) for the chemotherapy group. The median PFS was 4.5 months (95% CI: 4.2‐5.4 months) for the triplet‐therapy group, 4.3 months (95% CI: 4.1‐5.4 months) for the doublet‐therapy group and 1.5 months (95% CI: 1.5‐1.9 months) for the chemotherapy group^87^ (Table 1). In a real‐world study in Italy, the ORR for 36 patients treated with triplet‐target therapy was 31%, higher than the 17% observed in doublet‐therapy group (*p* = .012).^61^

In the BEACON trial, the incidence of adverse events for triplet‐target therapy and doublet‐target therapy was 65.8% and 57.4%, respectively, while the FOLFIRI group had a higher adverse event rate of 64.2%. Relative to doublet‐target therapy, triplet‐target therapy exhibited a higher incidence (>10.0% difference in frequency) of dermatitis acneiform, diarrhoea, constipation and vomiting.^99^ The results of the BEACON CRC trial fully confirmed the superior efficacy of targeted therapy against the MAPK pathway. In subgroup analysis, patients belonging to the BM1 subtype exhibited higher response rates, median PFS and OS, compared to patients in the BM2 subtype when treated with triplet therapy.^100^ Hence, in 2020, the doublet regimen received approval from both the FDA and the European Commission for use as the second‐line treatment of *BRAF* ^*V600E*^‐mutant mCRC patients who have failed first‐line therapies.

Compared to the efficacy of current first‐line treatment, both doublet and triplet therapies exhibited the potential to become first‐line therapeutic strategies. Recently, the result of a multicentre single‐arm trial (ANCHOR CRC) evaluating triplet‐target therapy for first‐line treatment in patients with *BRAF*‐mutant CRC showed an ORR of 47.4%, median PFS of 5.8 months, and median OS of 18.3 months, with no severe adverse events reported in the 95 enrolled patients.^66^ Although the result of the ANCHOR CRC trial is quite promising, this trial was not conducted with a randomised controlled design and still requires further confirmation.

### 3.3 Combination of chemotherapy and MAPK‐related targeted therapy

After exploring the use of MAPK pathway inhibitors in treating *BRAF* ^*V600E*^‐mutant mCRC patients, researchers have also attempted to combine chemotherapy with MAPK‐related inhibitors. In the Phase II prospective study (SWOG S1406), the combination of vemurafenib with irinotecan and cetuximab (VIC) achieved a disease control rate (DCR) of 65% in *BRAF^V600E^*‐mutant mCRC patients, while the irinotecan and cetuximab‐only group had a rate of 21%^69^ (Table 1). In a real‐world study conducted in China, patients receiving VIC as first‐line therapy demonstrated superior clinical outcomes compared to those undergoing conventional chemotherapy.^101^ Moreover, in the Phase II clinical trial (IMPROVEMENT), researchers utilised FOLFIRI in combination with vemurafenib and cetuximab for the second‐line treatment. The result of IMPROVEMENT showed that patients receiving this therapy experienced manageable adverse events, achieved an ORR of 81% and a median PFS of 9.7 months^70^ (Table 1). The findings from the mentioned clinical trials provide initial insights that the utilisation of chemotherapy alongside BRAF inhibitors demonstrates superior efficacy compared to standard chemotherapy plus bevacizumab, and even outperforms triplet‐targeted therapy.

For further validation, the regimen of chemotherapy + anti‐EGFR therapy + BRAF inhibitors is also being considered as the first‐line therapeutic strategy for CRC patients with *BRAF* ^*V600E*^ mutation. In studies using tumour xenografts, this approach has demonstrated superior anti‐tumour activity with a lower incidence of resistance compared to targeted therapy alone.^102^ Currently, the Phase III clinical trial (BREAKWATER) is investigating the effectiveness and tolerability of encorafenib + cetuximab + chemotherapy as a first‐line therapeutic approach for patients with *BRAF* ^*V600E*^‐mutant CRC. The results of safety lead‐in trial indicated that among 57 previously untreated patients who received treatment with encorafenib + cetuximab + FOLFIRI (*n* = 30) or encorafenib + cetuximab + mFOLFOX6 (*n* = 27), the incidence of severe (≥ grade 3) adverse events was 33% and 56%, respectively.^103^ In future, this trial plans to enrol 750 patients globally and employ a randomised and controlled method to validate these findings.^104^

### 3.4 Combination of MAPK‐ and PI3K/AKT‐related targeted therapy

As mentioned before, feedback activation of EGFR on the cell membrane could lead to the phosphorylation of PI3K. Preclinical researches have also revealed that CRC cell lines resistant to MEK inhibitors demonstrate minimal or no activation of ERK1/2 but show concurrent activation of the PI3K pathway.^105^ Besides, alterations in Phosphatase and Tensin Homolog (*PTEN*) are associated with *BRAF* mutations in CRC and melanomas.^106^, ^107^ Besides *PTEN*, Phosphatidylinositol‐4,5‐Bisphosphate 3‐Kinase Catalytic Subunit Alpha (*PIK3CA*) gene mutations occur in about 15% of all patients and are often detected alongside other gene mutations, including *BRAF*.^108^ Notably, activating mutations in *PIK3CA* or inactivating mutations in *PTEN* may significantly influence the effectiveness of anti‐EGFR therapy.^109^, ^110^ Therefore, the abnormal activity of the PI3K signalling, along with reactivation of MAPK signalling, may explain the modest efficacy observed in patients with *BRAF* ^*V600E*^‐mutant CRC treated with BRAF inhibitors alone.

In vitro experiments, inhibitors targeting AKT and mTOR can effectively enhance the cytotoxic effects of vemurafenib^111^ (Figure 1).  In a Phase Ib clinical trial, PI3K inhibitor (alpelisib) was included in the experimental regimen. The triplet usage of encorafenib + cetuximab + alpelisib demonstrated an ORR of 18% and a median PFS of 4.2 months in 28 patients with *BRAF* ^*V600E*^‐mutant mCRC who had failed first‐line treatment^67^ (Table 1). In the subsequent Phase II trial, the median PFS for 52 patients receiving the triplet therapy was 5.4 months. Although there was a slight improvement in efficacy compared to the dual therapy without alpelisib (HR = 0.69; 95% CI: 0.43‐1.11; *p* = .064), 79% of patients receiving triple therapy reported adverse events of grade 3 or higher, while the dual therapy group had a lower incidence at 58%^112^ (Table 1).

Considering alpelisib's pharmacological mechanism, this result suggests that inhibiting the activation of PI3KCA alone does not enhance the efficacy of BRAF‐targeted therapy significantly. Instead, it introduces additional side effects such as anaemia and hyperglycaemia. However, this clinical trial did not report mutations in *PTEN* and *PIK3CA* among the patients, precluding any subgroup analysis related to gene mutations in PI3K pathway.

### 3.5 Immunotherapy

Over the past decade, immune cells within tumour tissues have gained increasing attention and immune checkpoint inhibitors (ICIs) has shown significant efficacy in the treating various solid tumours.^113^ ICIs can improve the anti‐tumour immune response by inhibiting negative regulators on T cells, including Programmed Cell Death‐1 (PD‐1) inhibitors (e.g. pembrolizumab, nivolumab), and cytotoxic T‐lymphocyte‐associated protein 4 (CTLA‐4) inhibitors (e.g. ipilimumab) (Figure 2). In patients with MSI‐H CRC, treatment with PD‐1 blockade antibodies has demonstrated durable responses and control of the disease.^114^ As mentioned earlier, about 40% of *BRAF* ^*V600E*^‐mutant tumour tissues also exhibit MSI‐H. Likewise, according to the CMS subtype of CRC, the majority of *BRAF* ^*V600E*^‐mutant tumours belong to CMS1, also known as the MSI Immune subtype.^115^ Therefore, patients with *BRAF* ^*V600E*^ ‐mutant CRC may benefit from ICI therapy.

In the Phase III trial (KETNOTE‐177), 307 untreated mCRC patients with MSI‐H were randomly distributed into the chemotherapy group or the pembrolizumab group. Patients receiving ICI therapy had a median PFS of 16.5 months compared to 8.2 months in the standard chemotherapy group. The ICI group achieved a remarkable ORR of 83%, while only 35% of patients receiving chemotherapy experienced the regression of disease.^116^ Among the ICI and chemotherapy groups, 35 patients (23%) and 44 patients (29%), respectively, had *BRAF* ^*V600E*^ mutation. Subgroup analysis revealed that the presence of *BRAF* mutations did not significantly impact the efficacy of pembrolizumab monotherapy (*p* = .81) (Table 1).^71^ In the KEYNOTE‐164 trial, pembrolizumab monotherapy was administered as salvage treatment for MSI‐H CRC patients who had failed standard treatment. The ICI therapy still demonstrated an ORR of 33%, and among the 14 patients with *BRAF* ^*V600E*^ mutation in the cohort, the ORR was 42% (6/14)^72^ (Table 1). The results of these two trials suggest that pembrolizumab is the preferred treatment for both MSI‐H and *BRAF* ^*V600E*^‐mutant CRC.

In addition to using PD‐1 monotherapy, clinical trials combining two ICI therapies, CTLA‐4 and PD‐1, have also shown promising results. In the Phase II study (Checkmate‐142), low‐dose ipilimumab and nivolumab were used to treat MSI‐H mCRC patients who progressed after standard chemotherapy. Among the 119 patients, 25% harboured *BRAF* ^*V600E*^ mutation, and the 12‐month PFS rate was 71%.^117^ In the subsequent 4‐year follow‐up statistics, the ORR was 65%, complete response rate was 13%, and the median PFS had not been reached (95% CI: 38.4 months—not estimable), with a 32% occurrence of grade 3−4 therapy‐associated adverse events^73^ (Table 1). Similarly, patients with or without *BRAF* ^*V600E*^ mutations both responded well to this treatment. These results demonstrate that MSI‐H mCRC patients can tolerate and continue to benefit from anti‐CTLA‐4 and PD‐1 treatments, but the dual therapy also brought about stronger toxicity. The usage of low‐dose ipilimumab and nivolumab has also been considered for first‐line treatment in MSI‐H CRC patients. In a preliminary small‐sample trial (*n* = 45), 38% of patients had *BRAF* ^*V600E*^ mutations. This regimen showed an ORR of 69%, a 2‐year PFS rate of 74%, and a 22% incidence of grade 3−4 treatment‐induced adverse events^74^ (Table 1). This suggests that dual immune checkpoint therapy has the potential to be a first‐line treatment for MSI‐H and *BRAF* ^*V600E*^‐mutant mCRC patients but still requires large‐scale and direct comparison study.

### 3.6 Combination of immunotherapy and target therapy

In the murine model, the combined treatment of trametinib and PD‐1 monoclonal antibody demonstrated superior efficacy compared to the sole use of PD‐1 antibody. Meanwhile, the CD8^+^ T cell abundance within tumours was higher than that observed in mice treated with trametinib or PD‐1 antibody alone.^118^ Similarly, preclinical studies using murine model have found that the combined use of type II RAF inhibitors (BGB‐283 or RAF‐709) and MEK inhibitors leads to an increased number of CD8^+^ T cells and activated T cells subpopulation within the tumour. This therapy also induces tumour regression through CD8^+^ T cells.^119^ Hence, targeting the MAPK signalling pathway may enhance the immune response of tumour cells. Therefore, combining anti‐PD‐1 monoclonal with MAPK pathway inhibitors may achieve better therapeutic outcomes. To verify this hypothesis, an early‐stage trial using the regimen of PD‐1, BRAF, and MEK inhibitors for treating *BRAF* ^*V600E*^‐mutant CRC was designated, results indicated an ORR of 24.3% and a median PFS of 4.3 months (95% CI: 3.7‐7.3 months) in 37 patients, outperforming the dual‐target approach using BRAF and MEK inhibitors. In the microsatellite stable (MSS) subgroup (*n* = 28), the ORR also reached 25%, with a median PFS of 5 months (95% CI: 3.7‐7.4 months), and Five patients with MSS have already received treatment for over a year^75^ (Table 1).

In addition to the combination of PD‐1, BRAF and MEK inhibitors, researchers have explored the usage of ICIs + BRAF inhibitors + anti‐EGFR therapies for treating mCRC patients with *BRAF^V600E^* mutation. In the clinical trial of NCT04017650, 23 patients with treatment‐refractory mCRC harbouring *BRAF^V600E^* mutations and MSS received the triplet therapy, comprising encorafenib, cetuximab and nivolumab. The results demonstrated an ORR of 45% (95% CI: 23−68%), a DCR of 95% (95% CI: 75−100), with a median PFS of 7.3 months and a median OS of 11.4 months (Table 1). Importantly, there were no dose‐limiting toxicities reported.^76^ The response rate of this therapy underscores that robust suppression of the MAPK pathway may enhance the immune response rate of tumours, providing a novel therapeutic plan for patients with both MSS and *BRAF^V600E^* mCRC patients. A randomised and controlled Phase II clinical trial for this regimen is currently recruiting patients.

### 3.7 Locoregional interventions

Due to the propensity for recurrence in patients with *BRAF* mutation, there is currently no consensus on the appropriateness of actively pursuing locoregional interventional therapies (LRIs) like surgical R0/R1 resection or radiotherapy for recurrent case. A retrospective study from China analysed the efficacy of curative LRIs on the prognosis of mCRC with *BRAF* mutation. The findings indicated that LRIs were linked to better clinical results in patients with *BRAF^V600E^* mutation (adjusted HR: 0.46, 95% CI: 0.22‐0.98; *p* = .044). After undergoing LRIs, patients with *BRAF^V600E^* mutation and CRLM experienced a significantly extended median OS, compared with palliative treatments (42.4 versus 23.7 months; HR: 0.11, 95% CI: 0.01‐1.22; *p* = .030).^120^ Furthermore, for patients with *BRAF^V600E^*‐mutant CRLM, those with node‐negative primary tumours and Carcinoembryonic Antigen (CEA) ≤ 200 µg/L exhibited a lower probability of postoperative recurrence and enjoyed prolonged survival benefits.^121^ At present, there are prospective clinical trials aimed at assessing whether the administration of BRAF inhibitors as neoadjuvant targeted therapy can enhance the likelihood and efficacy of curative surgery for *BRAF^V600E^*‐mutant patients with unresectable mCRC^122^ (Table 2).

**TABLE 2:** Ongoing clinical trials targeting specifically BRAF‐mutant metastatic colorectal cancer (mCRC) retrieved through a systematic review process performed on 10 April 2024.

Line of therapy	Study ID Status Main country	Phase	Therapy component	Main criteria
First‐line trials (intensive cytotoxic therapy)	NCT02162563 (CAIRO5) Recruiting Dutch	III	‐FOLFOX/ FOLFIRI + bevacizumab ‐FOLFOXIRI + bevacizumab ‐FOLFOX/ FOLFIRI + panitumumab	‐ Initially unresectable CRC with liver metastasis ‐ Known mutation status of RAS and BRAF
First‐line trials (MAPK‐targeted inhibitors with cytotoxic therapy)	NCT04607421 BREAKWATER (recruiting) USA	III	‐Encorafenib + Cetuximab ± chemotherapy ‐Chemotherapy alone	‐ Metastatic CRC patients with *BRAF* ^*V600E*^ ‐ Patients must not receive any prior treatment
First‐line trials (immunotherapy)	NCT05217446 SEAMARK (recruiting) USA	II	‐Encorafenib + Cetuximab + pembrolizumab ‐Pembrolizumab	‐ Stage IV CRC patients with *BRAF* ^*V600E*^ and MSI‐H ‐ Not received any systemic treatment before for metastatic CRC
Second‐line trials (MAPK‐pathway‐targeted therapy) Second‐line trials (MAPK‐pathway‐targeted therapy)	NCT04800822 Active, not recruiting USA	I	‐PF‐07284892 (SHP2 inhibitor) ‐PF‐07284892 + binimetinib + encorafenib	‐ Patients with solid tumour harbouring *BRAF* ^*V600E*^ mutation ‐ CRC patients with *BRAF* ^*V600E*^ resistant to BRAFi plus EGFRi
	NCT06102902 Not yet recruiting USA	I	‐ZEN003694 (BET inhibitor) + Cetuximab + encorafenib	‐ Metastatic CRC patients with *BRAF* ^*V600E*^ ‐ Progressed after at least 1 prior systemic treatment
	NCT05743036 Recruiting USA	I/II	‐ZN‐c3 (WEE1inhibitor) + encorafenib + Cetuximab	‐ Metastatic CRC patients with *BRAF* ^*V600E*^ mutation ‐ Progressed after at least 1 previous systemic treatment
	NCT04892017 Recruiting USA	I/II	‐DCC‐3116 (ULK1/2 inhibitor) ± RAS/MAPK pathway inhibitors	‐ Metastatic solid tumour with a documented RAS, NF1 or RAF mutations ‐ Progressed after standard systemic treatment
	NCT05957367 Recruiting USA	I/II	‐DCC‐3116(ULK1/2 inhibitor) + Cetuximab + encorafenib	‐ CRC patients with *BRAF* ^*V600E*^ mutation ‐ Patients must have received at least 1 lines of prior systemic therapy ‐ Patients must not have received prior treatment with EGFR or BRAF inhibitors
	NCT05576896 Recruiting USA	II	‐Hydroxychloroquine + encorafenib + Cetuximab/panitumumab	‐ Stage IV CRC patients with *BRAF* ^*V600E*^ mutation ‐ Patients must have received at least 1 lines of prior systemic therapy ‐ Patients must not have any previous BRAF inhibitor therapy
	NCT05786924 Recruiting USA	I	BDTX‐4933 (Pan‐RAF inhibitor)	‐ Recurrent or advanced metastatic solid tumours with BRAF or RAS mutations ‐ Patients must receive at least 1 but no more than 2 prior lines of systemic therapy
	NCT05538130 Recruiting USA	I	ARRY‐134 (MEK inhibitor) + encorafenib	‐ Metastatic solid tumour with *BRAF* ^*600E*^ mutation ‐ Progressed after pervious treatment
	NCT04190628 Recruiting USA	I	ABM‐1310 (BRAF inhibitor) ± Cobimetinib (MEK inhibitor)	‐ Locally advanced or metastatic solid tumour with *BRAF* ^*V600E*^ mutation ‐ Progressed after at least 1 line of prior standard systemic therapy
	NCT05985954 Not yet recruiting USA	I	Ulixertinib (ERK1/2 inhibitor) + Cetuximab + encorafenib	‐ Unresectable or metastatic CRC with *BRAF* ^*V600E*^ mutation ‐ Priorly treated with BRAF therapy (not including regorafenib) and anti‐EGFR therapy (Cetuximab or panitumumab)
	NCT05275374 Not yet recruiting China	I/IIa	XP‐102 (RAF inhibitor) ± trametinib	‐ Advanced solid tumour patients with BRAF^V600^ mutation ‐ Progressed after at least 1 line of prior standard systemic therapy
	NCT05039177 HERKULES‐3 (recruiting) USA	Ib/II	ERAS‐007 (ERK inhibitor) + BRAF inhibitor + Cetuximab	‐ Metastatic tumour patients with *BRAF* ^*V600E*^ mutation ‐ Patients must not have any RAS, MEK or ERK inhibitor previously
	NCT05233332 Recruiting China	II	HL‐085 (MEK1/2 inhibitor) ± vemurafenib	‐ Metastatic CRC patients with *BRAF* ^*V600E*^ ‐ Progressed after at least 1 or more lines of prior standard systemic therapy
	NCT06008119 Not yet recruiting China	III	‐Tunlametinib (MEK1/2 inhibitor) + vemurafenib ‐Chemotherapy‐based treatment	‐ Metastatic CRC patients with *BRAF* ^*V600E*^ ‐ Progressed after at least 1 or more lines of prior standard systemic therapy
Second‐line trials (MAPK‐targeted inhibitors with cytotoxic therapy)	NCT05540951 Recruiting China	III	‐VIC regimen (vemurafenib, irinotecan and Cetuximab) ‐Bevacizumab plus chemotherapy	‐ Initially unresectable metastatic or local CRC patients with *BRAF* ^*V600E*^ and RAS wild‐type ‐ 6 months after previous systemic treatment or radiotherapy
Second‐line trials (immunotherapy)	NCT05019534 Recruiting China	I	‐Camrelizumab + vemurafenib + Cetuximab	‐ Patients with *BRAF* ^*V600E*^ and MSS, ‐ Prior treatment with at least one systemic treatment, not include Cetuximab ‐ Unresectable and/or metastatic CRC
	NCT05308446 Recruiting USA	II	‐Nivolumab + encorafenib + Cetuximab ‐Encorafenib + Cetuximab	‐ Unresectable and/or metastatic CRC ‐ Patients with *BRAF* ^*V600E*^ and MSS ‐ Progressed after pervious standard systemic treatment
	NCT03668431 Recruiting USA	II	‐Dabrafenib + trametinib + PDR001	‐ Metastatic CRC patients with *BRAF* ^*V600E*^ ‐ Progressed after pervious standard systemic treatment ‐ Not used MAPK‐related target therapy before
	NCT05963087 Not yet recruiting China	II	‐Tislelizumab + Cetuximab + dabrafenib	‐ Advanced CRC with *BRAF* ^*V600E*^ ‐ Progressed after pervious standard systemic treatment
	NCT04653480 APHRODITE Recruiting China	II	‐Surufatinib (RTK) + toripalimab + chemotherapy	‐ Metastatic CRC patients with RAS/BRAF mutation and MSS ‐ Progressed after pervious standard systemic treatment
	NCT05985109 Recruiting China	II	‐KN046 (PD‐L1/CTLA‐4 bispecific inhibitor) + regorafenib	‐ Metastatic CRC patients with *BRAF* ^*V600E*^ and MSS ‐ Progressed after treatment with cytotoxic regimens
Neoadjuvant therapy (MAPK‐pathway‐targeted therapy)	NCT05510895 (AIO‐KRK‐0420) Recruiting Germany	II	Neoadjuvant encorafenib + binimetinib + Cetuximab	‐ Unresected localised CRC patients (T^3‐4^N^+^M^0^) with *BRAF* ^*V600E*^ and MSS ‐ Patients must get untreated before
	NCT05706779 (NEORAF) Recruiting France	II	Encorafenib + Cetuximab	‐ Patients with colon and upper rectum adenocarcinoma without metastases and considered operable ‐ *BRAF* ^*V600E*^ and RAS wild‐type must be confirmed ‐ Previously untreated with RAF inhibitors or anti‐EGFR treatment
Surgical intervention	NCT05881746 (ARCLAMP) Not applicable China	/	‐Anatomical liver resection ‐Nonanatomic liver resection	‐ Histologically confirmed colorectal cancer initially resectable liver‐only metastases ‐ Suitable for anatomical liver and nonanatomic resection ‐ KRAS/NRAS/BRAF mutation or right‐sidedness ‐ not systemic treated before

FOLFOX, 5‐fluorouracil, leucovorin and oxaliplatin; FOLFIRI, 5‐fluorouracil, leucovorin and irinotecan; FOLFOXIRI, 5‐fluorouracil, leucovorin, oxaliplatin and irinotecan.

## 4 POTENTIAL THERAPEUTIC TARGETS OF *BRAF^V600E^*‐MUTANT CRC

Despite significant progress in the treatment response and survival benefits of first‐ and second‐line therapies for *BRAF* ^*V600E*^‐mutant CRC patients, therapeutic outcomes for *BRAF* ^*V600E*^‐mutant CRC patients remain inferior compared to targeted therapy for *BRAF* ^*V600E*^‐mutant melanoma. Tissue‐specific differences in EGFR expression contribute to different efficacy of treatment, with CRC originating from high EGFR‐expressing epithelial cells, while melanoma arises from EGFR nonexpressing neural crest cells.

In addition to feedback activation mechanisms, genetic mutations in the MAPK signalling pathway, such as *KRAS exon 2 p.G12D* and *KRAS exon 3 p.Q61H*, *BRAF* amplification, *MEK1 exon 2 p.K57T* can also contribute to the development of acquired resistance.^123^ With advancements in liquid biopsy and sequencing technologies, several research teams have analysed circulating tumour DNA (ctDNA) or tumour tissue from patients with acquired drug resistance to BRAF‐targeted therapy. Their work uncovered potential associations between acquired resistance in *BRAF*‐mutant patients and the formation of RAF dimers, DNA damage repair pathways, and several novel gene alterations, including *MYC*.^124^, ^125^ Some individuals also exhibited the simultaneous emergence of multiple resistance gene alterations.^126^

**FIGURE 4:**
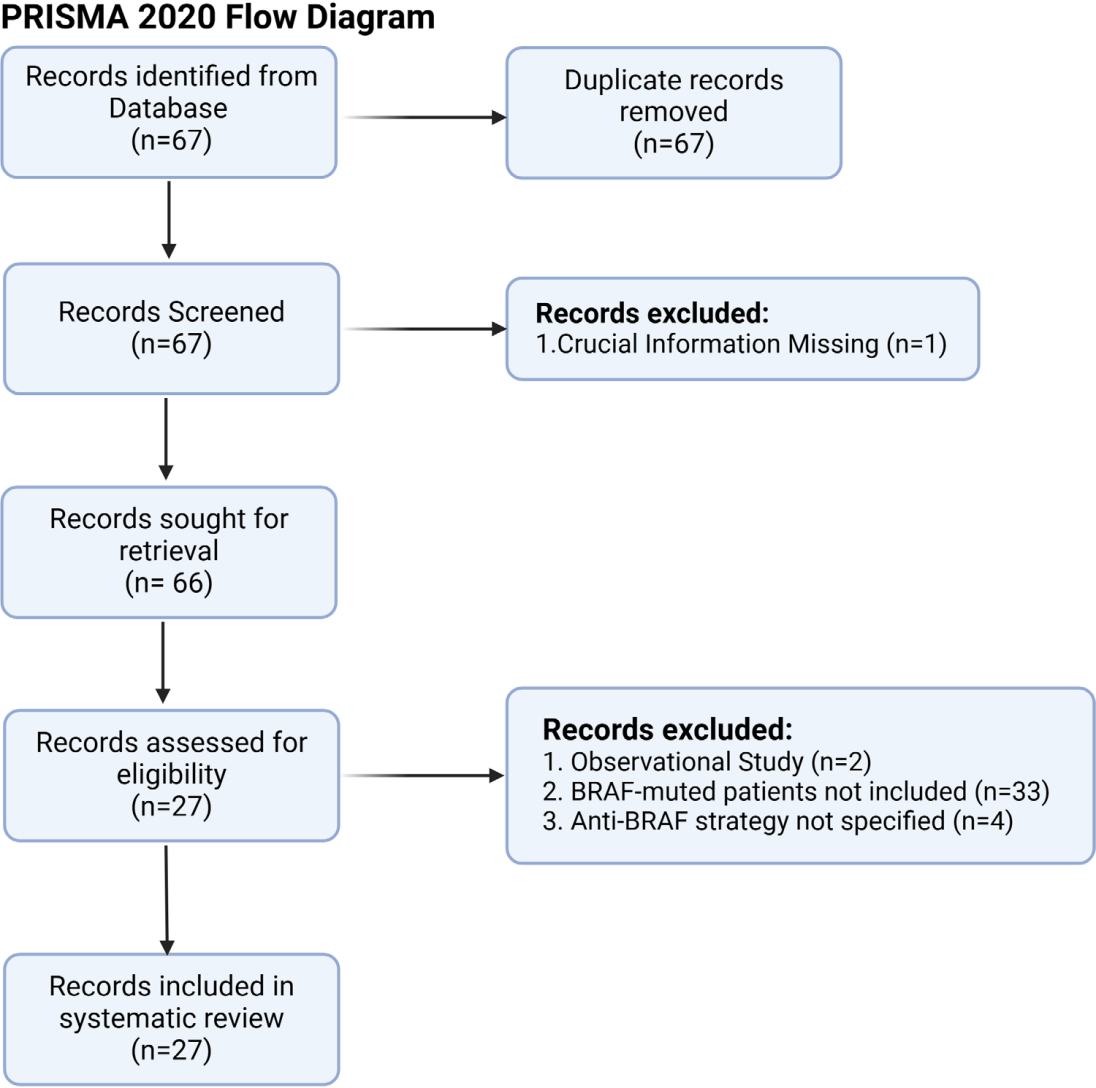
PRISMA 2020 Flow diagram illustrating the systematic review conducted on the data from ClinicaTrial.gov.

Based on preliminary basic research and preclinical studies, several novel therapeutic targets have been incorporated into the treatment strategies for *BRAF^V600E^*‐mutant CRC. In order to comprehensively assess potential therapeutic strategies for such patients, we conducted a systematic review of ongoing clinical trials on ClinicalTrials.gov in accordance with PRISMA guidelines.^127^ The Medical Subject Headings terms employed for the search in ClinicalTrials.gov were (‘Recruiting or not yet recruiting’ as status), (‘colo‐rectal cancer’ as condition/disease) and (‘*BRAF*’ as other terms). Two authors (R.G. and H.F.) independently conducted the systematic review process and three authors (W.D., R.W. and G.C.) checked the whole process. All ongoing studies who did not recruit *BRAF^V600E^*‐mutant patients were excluded (Figure 4).

To systematically illustrate potential targets for treating CRC patients with *BRAF^V600E^* mutation, we categorised these targets into the following five sections.

### 4.1 Targets associated with the MAPK pathway

Current target therapy for *BRAF^V600E^*‐mutant tumours primarily focuses on competitively binding to the cell membrane's EGFR and blocking the intracellular RAF‐MEK signalling pathway. Prior to the initiation of the cascade reactions in the MAPK pathway, Src Homology 2 Domain‐Containing Phosphatase 2 (SHP2) plays a crucial role in bridging extracellular signalling and intracellular cascades. Phosphorylated tyrosine residues on EGFR activate SHP2, anchoring it intracellularly and activating the downstream GRB2/SOS1 complex, ultimately enhancing RAS protein activity.^128^, ^129^ In addition, SHP2 can also regulate the activation of PI3K/AKT and JAK/STAT signalling pathways^130^ (Figure 2). Thus, the phosphorylation status of SHP2 critically shapes the activity of downstream effectors. Herr's research reported that the inhibition of BRAF leads to the upregulation of GRB2‐associated binders, Gab1 and Gab2, which is regulated by SHP2.^131^ Coincidentally, Morimoto's work explored that the oncogenic Cell‐Surface‐Associated Mucin 1 protein (MUC1‐C), revealing its critical role in driving BRAF‐inhibitor‐induced feedback of ERK signalling through the activation of SHP2^132^ (Figure 2). Therefore, SHP2 inhibitors have been developed to disrupt RAS‐GTP loading mediated by Son of Sevenless Homolog 1(SOS1) to reduce the activity of RAS/RAF/MEK/ERK signalling.^133^ In vitro studies targeting *BRAF* ^*V600E*^‐mutant CRC suggest that SHP2 inhibitors (TNO155) can enhance the ability of BRAF and MEK inhibitors to kill tumour cells^134^ (Table 2). At the moment, clinical trials are underway for the combination therapy of SHP2 molecular inhibitors (e.g. TNO155, PF‐07284892) with MAPK‐targeted drugs.

As previously mentioned, genetic alterations like *BRAF* amplification and fusion are common causes of acquired resistance to BRAF inhibitors in treating *BRAF^V600E^*‐mutant CRC.^135^ These genomic changes may lead to the reactivation of downstream molecules in the MAPK pathway through RAF protein dimerisation. Therefore, type II RAF inhibitors targeting RAF dimers may effectively reduce the occurrence of resistance to targeted therapy.^136^, ^137^ Type II RAF inhibitors, including AZ304 and LY3009120, have demonstrated superior anticancer effects in in vivo and in vitro models, compared to traditional BRAF monomer inhibitors like encorafenib.^138^, ^139^ However, the performance of LY3009120 in Phase I clinical trials has been less than satisfactory, with noticeable toxicity and limited tumour regression^137^ (Table 2).

In recent years, the ‘paradox breaker’ RAF inhibitors, exemplified by PLX8349, has shown promising clinical efficacy. These inhibitors can selectively suppress abnormal ERK signalling and feedback activation of EGFR driven by *BRAF*‐mutant dimers in tumour cells without affecting the function of RAF protein in normal cells.^140^ In Phase I/II clinical trials, 23% of patients with *BRAF^V600E^*‐mutant tumour who treated with PLX8349 exhibited PR.^141^ Moreover, a pioneering BRAF inhibitor, employing the technology of protein degradation‐targeted chimeras (PROTACs), has been developed and testified in vivo experiments, without inducing degradation in *BRAF* wild‐type cells^142^ (Table 2).In addition to bioengineered RAF inhibitors, some naturally occurring small molecules exhibit the capability to inhibit RAF‐RAS‐MEK activity. For instance, Wang's research uncovered that Erianin, isolated from orchidaceous plants, possesses inhibitory effects on the activity of CRAF and MEK1/2 in *BRAF^V600E^*‐mutant cell lines.^143^ Currently, preclinical studies targeting this small molecule are underway.

Located downstream of RAF proteins, the reactivation of ERK1/2 is a direct cause of acquired resistance to BRAF inhibitors. Therefore, various ERK1/2 inhibitors have also emerged.^144^ In the Phase I trial of the ERK1/2 inhibitor (Ulixertinib), around 14% of *BRAF^V600E^*‐mutant patients with advanced cancer demonstrated PR following the treatment. Subpopulations harbouring *BRAF* mutations could also benefit from Ulixertinib.^145^ Besides, researchers have also attempted to combine ERK1/2 inhibitors with MAPK‐pathway‐targeted therapy. An ongoing Phase I clinical study (NCT05985954) is now recruiting *BRAF^V600E^*‐mutant mCRC patients, to testify the safety and efficacy of Ulixertinib + cetuximab + encorafenib. In this trial, patients initiating dual therapy of encorafenib + cetuximab will also be intermittently given the ERK inhibitor (ERAS‐007) to prevent acquired resistance. In addition to Ulixertinib and ERAS‐007, ERK inhibitors such as LTT462, GDC‐0994 have also demonstrated promising single‐agent activity, and associated clinical trials have already completed patient recruitment (Table 2).^146^

Phosphorylated ERK1/2 has the capability to phosphorylate numerous downstream cytoplasmic and nuclear substrates, such as *myc* and *c‐fos*. Pranteda's research figured out that sustained expression of *myc* attenuates the cytotoxic effect induced by dabrafenib in *BRAF^V600E^*‐mutant CRC.^147^ Hence, directing interventions towards crucial transcription factors may represent a strategic approach for *BRAF^V600E^*‐mutant CRC. Among them, Bromodomain and Extra‐terminal domain (BET) protein family can selectively engage with acetylated histones, thereby contributing to the modulation of gene transcription.^148^ Inhibiting the bromodomain subunit of BET protein can disrupt the synthesis of the P‐TEFb complex, thereby downregulating the transcription of *myc* ^148^, ^149^ (Figure 2). Subsequently, researchers have developed various molecular inhibitors targeting this bromodomain structure. Given the reason that the acquired resistance to BET inhibitor may result from the overactivation of ERK, combining targeted therapy against MAPK pathway with BET inhibitors can effectively suppress the growth of tumour cell in vivo.^150^ In treating *BRAF^V600E^*‐mutant melanoma, intermittent BET inhibition prolongs response duration to the combination of BRAF + MEK inhibitors^151^ (Table 2). Similar conclusions have been confirmed in preclinical studies of *BRAF^V600E^*‐mutant CRC.^152^ Therefore, a Ib trial of the regimen combining BET inhibitor (ZEN003694) with cetuximab and encorafenib has been successfully proposed, with plans to enrol mCRC patients with *BRAF^V600E^* mutation.

### 4.2 Biological function mediated by MAPK pathway

In the study of acquired resistance mechanisms in tumour treatment, autophagy is acknowledged to promote tumour progression by digesting damaged organelles, thereby shielding cancer cells from the cytotoxic effects of chemotherapy.^153^ Similarly, in cells resistant to BRAF‐targeted therapy, autophagy often exhibits heightened activity. Several studies have now identified AMP‐activated protein kinase (AMPK) and ERK1/2 inhibition as factors that can enhance BRAF inhibitor‐induced autophagy.^154^, ^155^

In current research, unc‐51 like autophagy activating kinase 1/2 (ULK1/2) is considered a crucial link connecting the MAPK pathway and the cellular autophagic process. Activated ERK can phosphorylate liver kinase B1 (LKB1), facilitating its interaction with AMPK and subsequent activation of downstream ULK1/2, and then initiates cellular autophagy, which contributed to the acquired resistance against MAPK‐pathway‐targeted therapy^156^, ^157^, ^158^ (Figure 2). Therefore, inhibiting ULK1/2 may effectively decrease cellular autophagy. Preliminary results from a Phase I clinical trial of ULK1/2 inhibitor (DCC‐3116), presented at the 2022 ESMO poster exhibition, indicated that patients taking the inhibitor did not experience dose‐limiting toxic reactions and achieved the expected inhibition of ULK1/2.^159^ Two subsequent clinical trials, NCT05957367 and NCT04892017, are ongoing, where participants with *BRAF* mutation will receive treatment with DCC‐3116 in combination with MAPK‐pathway‐targeted drugs (such as encorafenib) to explore the efficacy of targeting both MAPK pathway and autophagy (Table 2).

Besides ULK1/2, hydroxychloroquine (HCQ), used in the treatment of lupus erythematosus, can also effectively block the activity of cellular autophagy. HCQ is a lysosomal inhibitor that can effectively inhibit Palmitoyl Protein Thioesterase 1 (PPT1), thereby inhibiting the binding of mTORC1 to lysosomes and the activation of autophagy^160^, ^161^ (Figure 2). In a Phase I/II trial for advanced *BRAF*‐mutant melanoma, the regimen of HCQ+dabrafenib+trametinib has demonstrated high ORR and safety.^162^ A Phase II clinical trial (NCT05576896) is currently underway for patients with *BRAF^V600E^*‐mutant CRC (Table 2).

As a crucial pathway for cell proliferation, the MAPK pathway regulates the expression of cell cycle‐related proteins such as cyclin D and CDK inhibitors, through MEK/ERK and transcription factors like AP1.^163^ In *BRAF^V600E^*‐mutant melanoma, increased MEK/ERK activity is accompanied by mutations or inactivation of cell‐cycle regulators like Cyclin‐dependent Kinase inhibitor 2A (*CDKN2A*) and Tumour Protein 53 (*TP53*).^107^ Similarly, in *BRAF^V600E^*‐mutant CRC, frequent comutations in negative cell cycle regulators like F‐Box and WD Repeat Domain Containing 7 (*FBXW7*) and *TP53* are observed, with mutation or deletion frequencies of 45% and 25%, respectively. These genetic changes lead to the amplification of downstream cell‐cycle‐related genes like *cyclin D* and *myc*.^164^ In addition, excessive activation of CDK1 was associated with the resistance to BRAF inhibitors in the treatment of CRC patients with *BRAF^V600E^* mutation^165^ (Figure 2).

As direct regulatory molecules of the cell cycle, targeted therapies against cyclin D and CDK4/6 have emerged. Selective CDK4/6 inhibitors can halt the progression of the tumour cell cycle.^166^ Therefore, the combination of MAPK pathway inhibitors and CDK4/6 inhibitors may be effective for certain subset of *BRAF*‐mutant CRC patients.^167^ In the clinical trial NCT02857270, some *BRAF*‐mutant mCRC patients are undergoing treatment with a combination of CDK4/6 inhibitor (Abemaciclib) and ERK1/2 inhibitor (LY3214996), and the results of this trial are pending publication (Table 2).

### 4.3 PI3K/AKT/mTOR pathway

Given the unsatisfactory results from the previous Phase II study, the role of this pathway in *BRAF^V600E^*‐mutant CRC remains controversial. Based on current researches, increased activity of the PI3K pathway or mutations in *PIK3CA* are closely associated with acquired resistance to BRAF inhibitors. Conversely, in the trial using dabrafenib and trametinib to treat *BRAF* ^*V600E*^‐mutant CRC, three patients with partial or complete responses had *PIK3CA* mutations.^65^ Subgroup analysis in the clinical trial using the VIC regimen also revealed that patients with *PI3KCA* mutations experienced more survival benefits than wild‐type patients on PFS (HR: 0.3 vs. 0.6).^69^ Thus, understanding these molecular intricacies is crucial for optimising treatment strategies and addressing the acquired resistance in advanced melanoma and CRC with *BRAF^V600E^* mutation.

### 4.4 Wnt/β‐catenin pathway

Based on previous findings, attempts to combine Wnt inhibitors with MAPK‐targeted therapy are underway, aiming to reduce the incidence of acquired drug resistance.^168^ However, initial results from a Phase Ib clinical trial (NCT02278133) involving the Wnt inhibitor (WNT974), encorafenib, and cetuximab showed considerable toxicity, with 75% of patients experiencing severe adverse events such as bone pain and fractures, while achieving only an ORR of 10%.^169^ Due to these significant orthopaedic complications, subsequent Phase II trials have been cancelled.

Despite the less‐than‐ideal outcomes of Wnt inhibitor combination with BRAF inhibitors, Researchers are investigating the prognostic implications of certain Wnt pathway negative regulators, such as *RNF43* mutations and R‐Spondin (*RSPO*) fusions. *RNF43* mutations and *RSPO* fusions result in the overexpression of Wnt receptors on the cell membrane, leading to excessive activation of the Wnt pathway. Thus, patients with *RNF43* mutations and *RSPO* fusion CRC generally have a poorer prognosis. However, in the MSS subtype of *BRAF^V600E^*‐mutant CRC with *RNF43* mutations, patients exhibit a more favourable prognosis compared to those with wild‐type *RNF43*.^170^, ^171^, ^172^ Elena et al. also discovered that in the MSS subtype of *BRAF^V600E^*‐mutant patients with mutated *RNF43*, the ORR of doublet target therapy (encorafenib +cetuximab) was 63%. The sensitivity for predicting doublet treatment response using *RNF43* mutations is 60%, with a specificity of 72% in MSS subgroup (Figure 2). In a subsequent validation cohort, MSS and *RNF43*‐mutant patients receiving dual‐targeted therapy with *BRAF* and EGFR had a median PFS of 10.1 months, much higher than the 4.1 months for MSS and *RNF43* wild‐type patients.^172^ This result was also confirmed by a real‐world study.^173^

This nuanced finding highlights the intricate nature of the Wnt/β‐catenin pathway in *BRAF^V600E^*‐mutant CRC, warranting further experimental studies to elucidate underlying mechanisms (Figure 2).

### 4.5 Other therapeutic targets

Apart from the signalling pathways closely associated with *BRAF^V600E^*, researchers have identified specific molecule may contribute to the acquired resistance to BRAF inhibitors. In a recent study by Ruiz‐Saenz, it was found that BRAF inhibitors could activate SRC proto‐oncogene (SRC), and activated SRC kinase led to resistance to BRAF inhibitors. Coincidentally, increased activity of SRC has also been observed in EGFR inhibitor‐resistant patient‐derived xenograft (PDX) models.^174^ SRC is a group of nonreceptor tyrosine kinases that can interact with EGFR receptors, leading to the autophosphorylation of EGFR.^175^ The mechanism analysis revealed that activated SRC could reprogram the transcriptional activity of β‐catenin through phosphorylation, mediating the downstream gene expression and thus mediating resistance to BRAF inhibitors in *BRAF^V600E^*‐mutant CRC. SRC is not a primary driver of tumourigenesis, leading to limited efficacy in monotherapy targeting.^176^ Ruiz‐Saenz's team also discovered that using Celecoxib to inhibit Cyclooxygenase‐2(COX2), which is the upstream of SRC, could continuously mitigating the acquired resistance^177^ (Figure 2). Those preclinical studies emphasised the significance of SRC in acquired resistance to MAPK‐targeted therapy. In the future, inhibition of SRC may warrant consideration in the clinical trial who are recruiting patients resistant to BRAF inhibitors.

In addition to SRC family, in vitro experiments conducted by Erika et al. revealed that approximately 30% of CRC cells, encompassing alterations in common genes such as *RAS*, *BRAF* and others, responded to treatment with inhibitors targeting the DNA damage response (DDR) pathway.^178^ The presence of *BRAF^V600E^* accelerates the proliferation rate of CRC cells, while the absence or inhibition of the DDR pathway may aid cancer cells in evading regulatory checkpoints in the cell cycle, allowing entry into the mitotic phase. Although there is currently no clinical method to detect overall changes in DDR pathways, MMR, a component of the DDR pathway, is routinely tested in CRC patients. There are several methods to detect patient's MMR status, including immunohistochemistry (IHC) testing, polymerase chain reaction (PCR) methods and next generation sequencing (NGS). IHC testing is commonly used by pathologists to determine a patient's MMR status due to its affordability and accessibility. However, IHC testing methods can produce both false‐positive and false‐negative results. A patient with mCRC had a benign germline MutL homolog 1 (*MLH1*) variant, *MLH1 p.V384D*, and was reported to have loss expression of MLH1 by IHC testing. However, subsequent NGS testing revealed the patient's MSS status, overturning the false‐positive diagnosis by IHC.^179^ It has also been reported that patients with germline frameshift mutation in *MSH2* experience premature termination of protein translation. Surprisingly, IHC test could yield false‐negative diagnoses in these cases.^180^ These false‐negative and false‐positive results suggest that relying solely on IHC testing may not be sufficiently reliable. According to the NCCN guideline and the College of American Pathologists guideline, while IHC or PCR testing is the preferred method for assessing MMR status, additional testing should be conducted to confirm the patient's MMR status and prevent false‐positive results from IHC.^79^, ^181^

As one of crucial proteins in DDR and cell cycle checkpoint of G2/M phase, Wee1‐like protein kinase (WEE1) is located in the cell nucleus normally.^182^ However, in tumour cells, WEE1 functions as an oncogene, leading to the tolerance of genomic instability and fostering proliferation^178^, ^183^ (Figure 2). Currently, the small‐molecular WEE1 inhibitor (ZN‐c3) has demonstrated good safety in early‐phase clinical trials, with 17% of participants with various tumours exhibiting PR to this inhibitor.^184^ Consequently, researchers are exploring the combination of ZN‐c3 with encorafenib and cetuximab (NCT05743036), and this study is currently enrolling patients with *BRAF^V600E^*‐mutant mCRC (Table 2).

Based on previous researches, Mooradian's team conducted a Phase I clinical trial using a combination therapy of small molecule HSP90 inhibitor (AT13387) in conjunction with dabrafenib and trametinib. The ORR of this regimen for 22 patients with metastatic *BRAF^V600E^*‐mutant solid tumours was only 9.5%. Further investigation is warranted to identify the patients that may benefit from this regimen^185^ (Figure 2).

## 5 NOVEL FINDINGS AWAITING CLINICAL TRANSLATION

### 5.1 Intestinal epithelial dedifferentiation

Patients diagnosed with serrated CRC are found to have an increased prevalence of *BRAF^V600E^* mutation. Multiple studies employing patient tissues or organoid have also confirmed that reduced tissue differentiation is a characteristic feature of *BRAF^V600E^‐*driven CRC and serrated adenoma.^186^, ^187^ As reported, caudal type homeobox 2 (CDX2) and SMAD family member 4 (SMAD4) function as crucial transcription factor of intestinal differentiation. Research indicates a concomitant occurrence of *BRAF^V600E^* mutation and loss function of *CDX2* and *SMAD4* in serrated CRC tissues and cell lines. The loss of *CDX2* may result in diminished differentiation of intestinal epithelium. Likewise, the inhibition of *BRAF^V600E^* not only leads to an upregulation of CDX2 expression but also induces the formation of glandular structures in the epithelial.^188^, ^189^ The interplay between these genetic alterations collaboratively promotes several oncogenic pathways, actively driving serrated colorectal tumourigenesis in vivo^190^ (Figure 3A). As pivotal transcriptional regulators, both CDX2 and SMAD4 demand cautious consideration in drug development to avoid interfering with the differentiation and apoptosis of normal intestinal cells.

### 5.2 Nutrient metabolism

Similar to other tumours, cells harbouring the *BRAF^V600E^* mutation underwent metabolic reprogramming. Enhanced glucose uptake, glycolysis, cholesterol biosynthesis and glutamine metabolism are observed in *BRAF^V600E^*‐mutant cell lines and mouse model.^191^, ^192^ Yukimoto's work demonstrated that the upregulation of enolase 2 (ENO2), a glycolytic enzyme, is regulated by ERK in *BRAF^V600E^*‐mutant CRC. Inhibition of ENO2 led to increased sensitivity to vemurafenib, accompanied by concurrent suppression of both PI3K/AKT and MAPK pathways.^193^ Besides, *BRAF^V600E^*‐mutant CRC exhibit elevated levels of glutamate‐cysteine ligase (GCL), a rate‐limiting enzyme in the synthesis of glutathione. Heightened GCL expression protects *BRAF^V600E^*‐mutant tumours from oxidative stress during the metastatic process, particularly as they spread to distant organs such as the liver and lungs.^194^ However, the delicate metabolic pathways in tumour cells and challenges in preserving normal cellular metabolism impede the clinical translation of these findings.

### 5.3 Gut microbiota

Patients with the *BRAF^V600E^* mutation exhibit a distinct intestinal microbiota composition. *Prevotella enoeca* demonstrates higher abundance in patients with *BRAF^V600E^* mutation, while *Ruthenibacterium lactatiformans* exhibits increased prevalence in the gut microbiota of patients with wild‐type *BRAF*. The abundance of these two bacteria effectively distinguishes the status of the *BRAF* gene in patients^195^ (Figure 3A). This discovery may offer a novel noninvasive diagnostic approach for *BRAF* mutations. Furthermore, the interplay between *BRAF* mutation and *enterotoxigenic Bacteroides fragilis* has the potential to induce the CRC model in Lgr5^Cre^Min mice, resembling *BRAF^V600E^*‐driven CRC in human. This outcome substantiates the notion that genetic and microbiota interactions may play a role in the onset of *BRAF^V600E^* mutation‐driven CRC.^196^ Currently, researches on the interplay between gut microbiota and *BRAF*‐mutant CRC is still in early stage. The dynamic fluctuations and individual variances of gut microbiota need further investigation.

## 6 CONCLUSION AND PERSPECTIVES

CRC is among the most prevalent tumours worldwide. Thanks to the advancements in immunotherapy and target therapy, the prognosis for many CRC patients has improved notably. However, patients with the *BRAF^V600E^* mutation have not experienced remarkable benefits from these therapeutic advancements.

**FIGURE 5:**
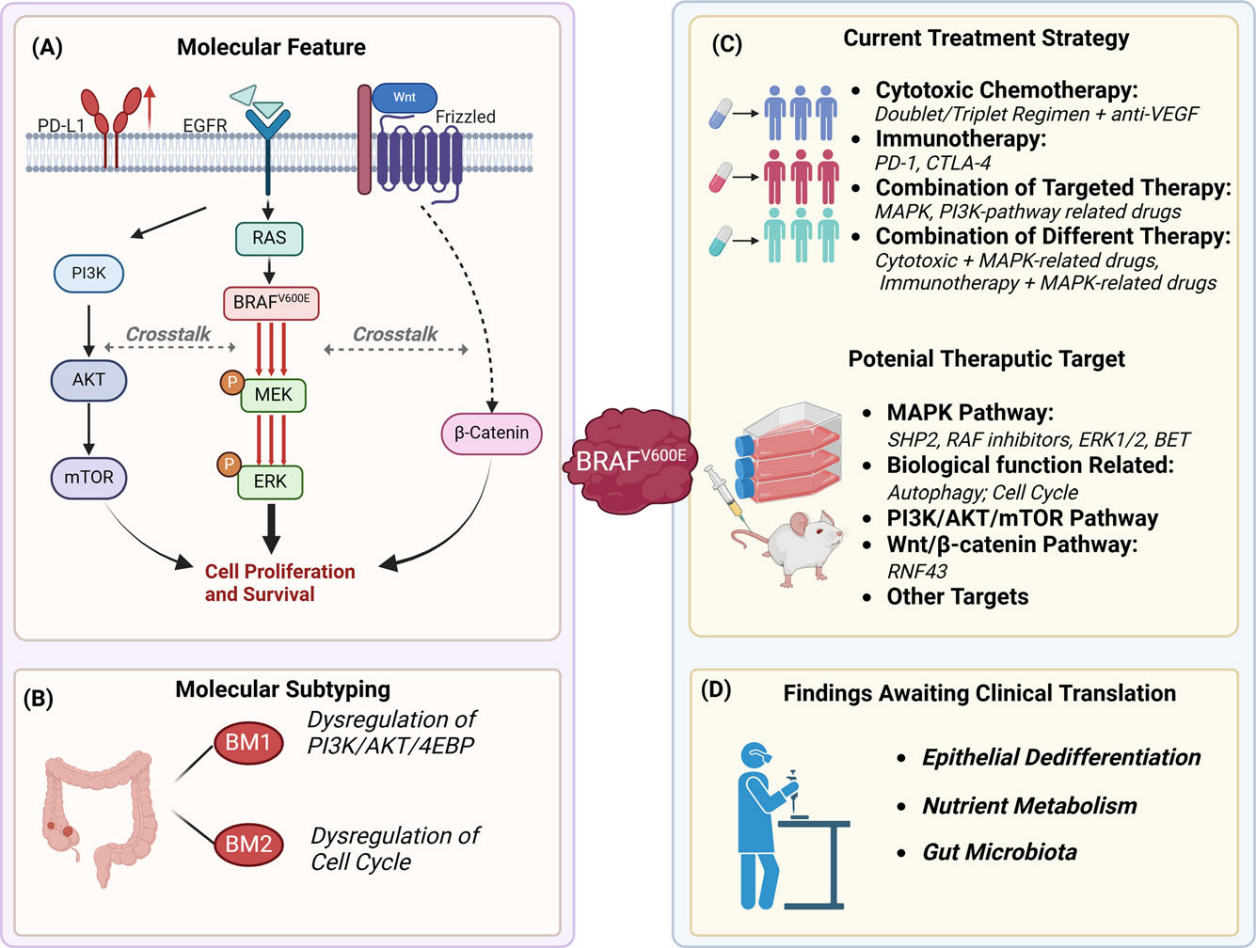
The schematic illustration demonstrates the molecular characteristics and therapeutic approaches in CRC with *BRAF^V600E^* mutation. (A) The foundational molecular and signal pathway alterations in *BRAF* ^*V600E*^‐mutant CRC. (B) Molecular subtyping in CRC patients with *BRAF^V600E^* mutation. (C) The current treatment strategy and potential therapeutic target in treating CRC patients with *BRAF^V600E^* mutation. (D) Novel research findings in the bench awaiting the clinical translation.

Therefore, clinicians and scientists have conducted many foundational investigations into the molecular pathology of this specific subtype and the effectiveness of various therapeutic drug combinations. Therapies targeting the MAPK pathway have demonstrated significant success in the second‐line treatment of *BRAF^V600E^*‐mutant CRC and have great potential to become first‐line treatment options. However, acquired resistance to targeted therapy continues to pose challenges for patients and researchers. Based on current research, reactivation of the MAPK pathway and other signalling pathways promoting cancer progression are crucial factors, contributing to acquired drug resistance in patients. Ongoing studies and clinical trials targeting these pathways are underway (Figure 5).

Moreover, beyond categorising patients with *BRAF^V600E^* mutation into subtypes BM1 and BM2, based on its transcriptomic features, there is a pressing need for in‐depth investigations into the metabolic alterations, protein translation and posttranslational modifications induced by *BRAF* mutations. Fundamental research in these areas holds significant promise for informing therapeutic decisions and advancing translational studies for *BRAF*‐mutant patients. In the future, unravelling the complexities of drug resistance mechanisms and developing effective therapeutic strategies are still the keys to improve the clinical outcomes of *BRAF^V600E^*‐mutant CRC patients and during the design of clinical trials, efforts should be made to integrate molecular information, such as BM1, BM2, CMS classification, aiming for precision treatment strategies.

## AUTHOR CONTRIBUTIONS

All authors contributed to the study conception and design. CGX and WRJ designed the work. GRQ, FHS and DWX drafted and revised the manuscript. All authors approved this manuscript.

## CONFLICT OF INTEREST STATEMENT

The authors declare that they have no competing interests.

## ETHICS STATEMENT

This is a review article and this article is not related to the approval of ethics.

## CONSENT FOR PUBLICATION

All authors agree with the content of the review and consent to publication.

## Data Availability

Information about the ongoing clinical trials can be found in www.ClinicalTrials.gov.
